# Diethyl 2,2-bis­(3,5-di-*tert*-butyl-4-hy­droxy­benz­yl)malonate

**DOI:** 10.1107/S1600536811054900

**Published:** 2012-01-18

**Authors:** Tao Zeng, Yu-Ping Hou, Wan-Zhong Ren, Wen-You Xu

**Affiliations:** a Chemistry & Biology College, Yantai University, Yantai 264005, People’s Republic of China; b Yantai University, Yantai 264005, People’s Republic of China

## Abstract

The title mol­ecule, C_37_H_56_O_6_, possesses twofold symmetry, with the twofold axis passing through the quaternary C atom. In the crystal, neighbouring mol­ecules are linked *via* O—H⋯O hydrogen bonds involving the phenol OH group and the carbonyl O atom, forming chains propagating in [101]. Within these chains, rings are formed with an *R*_2_^2^(20) motif. There are also C—H⋯O inter­actions present within the rings.

## Related literature

For hindered phenol anti­oxidants and their applications, see: Eggensperger *et al.* (1974 ▶, 1976 ▶); Breese *et al.* 2000 ▶; Yamazaki & Seguchi (1997 ▶). For the synthesis of hindered phenol anti­oxidants, see: Eggensperger *et al.* (1974 ▶, 1976 ▶). For hydrogen-bond motifs, see: Bernstein *et al.* (1995 ▶).
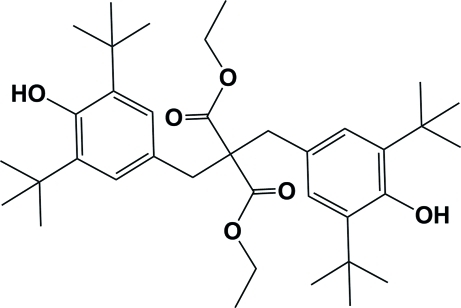


## Experimental

### 

#### Crystal data

C_37_H_56_O_6_
*M* *_r_* = 596.82
Monoclinic, 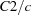

*a* = 20.006 (6) Å
*b* = 13.610 (4) Å
*c* = 14.252 (4) Å
β = 111.344 (5)°
*V* = 3614.4 (18) Å^3^
*Z* = 4
Mo *K*α radiation
μ = 0.07 mm^−1^
*T* = 294 K
0.22 × 0.18 × 0.16 mm

#### Data collection

Bruker SMART 1000 diffractometer
Absorption correction: multi-scan (*SADABS*; Sheldrick, 1996 ▶) *T*_min_ = 0.984, *T*_max_ = 0.989
9457 measured reflections
3280 independent reflections
2010 reflections with *I* > 2σ(*I*)
*R*_int_ = 0.042

#### Refinement

*R*[*F* ^2^ > 2σ(*F* ^2^)] = 0.053
*wR*(*F* ^2^) = 0.151
*S* = 1.06
3280 reflections
206 parameters
1 restraint
H atoms treated by a mixture of independent and constrained refinement
Δρ_max_ = 0.34 e Å^−3^
Δρ_min_ = −0.25 e Å^−3^

### 

Data collection: *SMART* (Bruker, 1997 ▶); cell refinement: *SAINT* (Bruker, 1997 ▶); data reduction: *SAINT*; program(s) used to solve structure: *SHELXS97* (Sheldrick, 2008 ▶); program(s) used to refine structure: *SHELXL97* (Sheldrick, 2008 ▶); molecular graphics: *PLATON* (Spek, 2009 ▶) and *Mercury* (Macrae *et al.*, 2008 ▶); software used to prepare material for publication: *SHELXL97* and *publCIF* (Westrip, 2010 ▶).

## Supplementary Material

Crystal structure: contains datablock(s) I, global. DOI: 10.1107/S1600536811054900/fb2240sup1.cif

Structure factors: contains datablock(s) I. DOI: 10.1107/S1600536811054900/fb2240Isup2.hkl

Supplementary material file. DOI: 10.1107/S1600536811054900/fb2240Isup3.cml

Additional supplementary materials:  crystallographic information; 3D view; checkCIF report

## Figures and Tables

**Table 1:** Hydrogen-bond geometry (Å, °)

*D*—H⋯*A*	*D*—H	H⋯*A*	*D*⋯*A*	*D*—H⋯*A*
O1—H1⋯O2^i^	0.80 (2)	2.38 (2)	2.996 (2)	134 (2)
C12—H12*C*⋯O2^i^	0.96	2.46	3.398 (3)	167

Symmetry code: (i) 
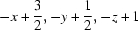
.

**Fig. 1.**
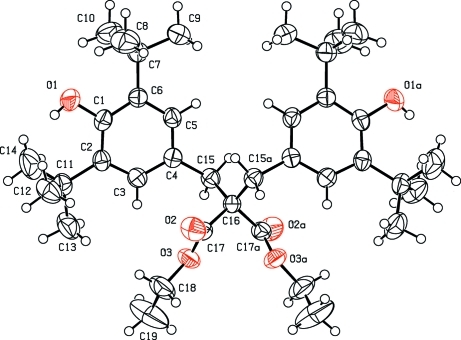
A view of the molecular structure of the title molecule, with the atomic numbering scheme and displacement ellipsoids drawn at the 50% probability level. The 2-fold axis bisects atom C16 [symmetry code: (a) -x+1, y, -z+1/2].

**Fig. 2.**
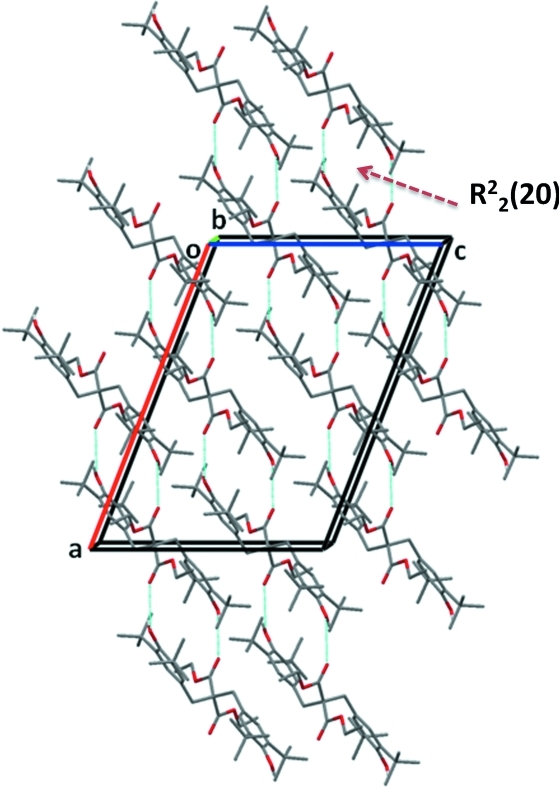
The crystal packing of the title compound, viewed along the b axis, showing the O—H···O hydrogen bonds and as dashed cyan lines [C-bound H atoms have been omitted for clarity; see Tab. 1 for details].

## supplementary crystallographic information

### Comment

A series of compounds containing the 2,6-di-*tert*-butylphenol moiety have been used in both polymers and lubricants as antioxidants (Eggensperger *et al.*, 1974, 1976). They are often called hindered phenol antioxidants due to the presence of the extremely large 2,6-di-*tert*-butylphenol moiety (Breese *et al.*, 2000; Yamazaki & Seguchi, 1997). As part of our ongoing studies of the chemistry of such compounds, we present herein the synthesis and the crystal structure of the title compound.

The title molecule is located about a 2-fold axis (Fig. 1), which passes through the quaternary carbon atom, C16. The bond angles involving C16 vary from 106.2 (2) to 111.9 (2)°. The remainder of the geometric parameters are within normal ranges.

In the crystal, neighbouring molecules are linked by O—H···O hydrogen bonds (Tab. 1 and Fig. 2), involving the phenolic OH group and the carbonyl O atom, forming chains in direction [1 0 1] that contain rings with a R^2^_2_(20) motif (Bernstein *et al.*, 1995). These rings are situated about the i nversion centres (the Wyckoff position 4c). C-H···O interactions are also present within the rings (Tab. 1).

### Experimental

A mixture of diethyl malonate (4.0 g, 0.025 mol) and 2,6-di-*tert*-butyl-4-((dimethylamino)methyl)phenol (13.2 g, 0.05 mol) in 80 ml of toluene was heated to 373 K under a nitrogen atmosphere. Lithium amide (0.2 g) was then added and the mixture was stirred for further 6 h. The reaction mixture was then diluted with toluene (60 ml), washed with water (40 ml), and the oil layer was separated and dried with anhydrous MgSO_4_. After filtration and concentration, the solution was allowed to evaporate in the air to give the title product as a white powder [yield 13.1 g; 87.9%]. Block-like colourless crystals with dimensions at about 2-3 mm were obtained by slow evaporation of the solution of the title compound in toluene/ethanol (3:1; v:v).

### Refinement

All the H-atoms could be located in difference Fourier maps. The hydroxyl H-atom was refined with a distance restraint of O—H = 0.82 (2) Å, and *U*_iso_H = 1.2*U*_eq(_O). The C-bound H-atoms were included in calculated positions and refined as riding atoms: C-H = 0.93, 0.97 and 0.96 Å for CH, CH_2_ and CH_3_ H-atoms, respectively, with U_iso_(H) = k × U_eq_(parent C-atom), where k = 1.5 for CH_3_ H-atoms and k = 1.2 for all other H-atoms.

### Crystal data

**Table:**

C_37_H_56_O_6_	*F*(000) = 1304
*M**_r_* = 596.82	*D*_x_ = 1.097 Mg m^−^^3^
Monoclinic, *C*2/*c*	Mo *K*α radiation, λ = 0.71073 Å
Hall symbol: -C 2yc	Cell parameters from 2211 reflections
*a* = 20.006 (6) Å	θ = 2.2–23.3°
*b* = 13.610 (4) Å	µ = 0.07 mm^−^^1^
*c* = 14.252 (4) Å	*T* = 294 K
β = 111.344 (5)°	Block, colourless
*V* = 3614.4 (18) Å^3^	0.22 × 0.18 × 0.16 mm
*Z* = 4	

### Data collection

**Table:**

Bruker SMART 1000 diffractometer	3280 independent reflections
Radiation source: fine-focus sealed tube	2010 reflections with *I* > 2σ(*I*)
graphite	*R*_int_ = 0.042
φ and ω scans	θ_max_ = 25.3°, θ_min_ = 1.9°
Absorption correction: multi-scan (*SADABS*; Sheldrick, 1996)	*h* = −24→20
*T*_min_ = 0.984, *T*_max_ = 0.989	*k* = −16→11
9457 measured reflections	*l* = −14→17

### Refinement

**Table:**

Refinement on *F*^2^	Secondary atom site location: difference Fourier map
Least-squares matrix: full	Hydrogen site location: difference Fourier map
*R*[*F*^2^ > 2σ(*F*^2^)] = 0.053	H atoms treated by a mixture of independent and constrained refinement
*wR*(*F*^2^) = 0.151	*w* = 1/[σ^2^(*F*_o_^2^) + (0.0716*P*)^2^ + 0.7136*P*] where *P* = (*F*_o_^2^ + 2*F*_c_^2^)/3
*S* = 1.06	(Δ/σ)_max_ < 0.001
3280 reflections	Δρ_max_ = 0.34 e Å^−^^3^
206 parameters	Δρ_min_ = −0.24 e Å^−^^3^
1 restraint	Extinction correction: *SHELXL97* (Sheldrick, 2008), Fc^*^=kFc[1+0.001xFc^2^λ^3^/sin(2θ)]^-1/4^
102 constraints	Extinction coefficient: 0.009 (1)
Primary atom site location: structure-invariant direct methods	

### Special details

**Table:**

Geometry. All esds (except the esd in the dihedral angle between two l.s. planes) are estimated using the full covariance matrix. The cell esds are taken into account individually in the estimation of esds in distances, angles and torsion angles; correlations between esds in cell parameters are only used when they are defined by crystal symmetry. An approximate (isotropic) treatment of cell esds is used for estimating esds involving l.s. planes.
Refinement. Refinement of F^2^ against ALL reflections. The weighted R-factor wR and goodness of fit S are based on F^2^, conventional R-factors R are based on F, with F set to zero for negative F^2^. The threshold expression of F^2^ > 2sigma(F^2^) is used only for calculating R-factors(gt) etc. and is not relevant to the choice of reflections for refinement. R-factors based on F^2^ are statistically about twice as large as those based on F, and R- factors based on ALL data will be even larger.

### Fractional atomic coordinates and isotropic or equivalent isotropic displacement parameters (Å^2^)

**Table:**

	*x*	*y*	*z*	*U*_iso_*/*U*_eq_	
O1	0.71866 (9)	0.42187 (11)	0.62113 (12)	0.0666 (5)	
H1	0.7512 (12)	0.3850 (16)	0.6488 (18)	0.080*	
O2	0.62147 (8)	0.10154 (10)	0.29387 (12)	0.0648 (5)	
O3	0.54675 (8)	0.02822 (10)	0.35618 (12)	0.0569 (5)	
C1	0.66141 (11)	0.37176 (15)	0.55316 (15)	0.0447 (5)	
C2	0.64934 (10)	0.27165 (15)	0.56606 (14)	0.0427 (5)	
C3	0.59129 (11)	0.22877 (15)	0.49092 (14)	0.0442 (5)	
H3A	0.5820	0.1627	0.4976	0.053*	
C4	0.54664 (10)	0.27825 (14)	0.40700 (14)	0.0410 (5)	
C5	0.55917 (10)	0.37794 (14)	0.40102 (15)	0.0442 (5)	
H5A	0.5288	0.4130	0.3460	0.053*	
C6	0.61499 (11)	0.42780 (14)	0.47341 (14)	0.0433 (5)	
C7	0.62391 (12)	0.54031 (16)	0.46818 (17)	0.0563 (6)	
C8	0.69596 (16)	0.56725 (19)	0.4609 (2)	0.0836 (9)	
H8A	0.6997	0.6374	0.4579	0.125*	
H8B	0.6992	0.5386	0.4011	0.125*	
H8C	0.7342	0.5427	0.5189	0.125*	
C9	0.56543 (16)	0.58469 (17)	0.3764 (2)	0.0934 (10)	
H9A	0.5193	0.5717	0.3801	0.140*	
H9B	0.5677	0.5559	0.3162	0.140*	
H9C	0.5725	0.6544	0.3753	0.140*	
C10	0.61797 (15)	0.58785 (18)	0.5627 (2)	0.0780 (8)	
H10A	0.5727	0.5708	0.5673	0.117*	
H10B	0.6213	0.6580	0.5584	0.117*	
H10C	0.6562	0.5645	0.6215	0.117*	
C11	0.69699 (12)	0.21224 (15)	0.65805 (15)	0.0509 (6)	
C12	0.77149 (12)	0.19636 (19)	0.6533 (2)	0.0744 (8)	
H12A	0.7666	0.1663	0.5902	0.112*	
H12B	0.7992	0.1543	0.7076	0.112*	
H12C	0.7953	0.2585	0.6590	0.112*	
C13	0.66642 (15)	0.10925 (19)	0.6605 (2)	0.0815 (9)	
H13A	0.6176	0.1147	0.6563	0.122*	
H13B	0.6944	0.0771	0.7223	0.122*	
H13C	0.6682	0.0716	0.6046	0.122*	
C14	0.7024 (2)	0.2628 (2)	0.75627 (18)	0.1034 (11)	
H14A	0.7281	0.3236	0.7627	0.155*	
H14B	0.7276	0.2208	0.8121	0.155*	
H14C	0.6551	0.2757	0.7557	0.155*	
C15	0.48315 (10)	0.22752 (14)	0.32896 (15)	0.0432 (5)	
H15A	0.4486	0.2774	0.2933	0.052*	
H15B	0.4604	0.1860	0.3639	0.052*	
C16	0.5000	0.16358 (19)	0.2500	0.0390 (7)	
C17	0.56390 (11)	0.09625 (14)	0.30121 (16)	0.0452 (5)	
C18	0.60223 (15)	−0.04431 (19)	0.4048 (2)	0.0806 (9)	
H18A	0.6138	−0.0811	0.3544	0.097*	
H18B	0.6454	−0.0117	0.4487	0.097*	
C19	0.5756 (2)	−0.1098 (2)	0.4627 (3)	0.1349 (15)	
H19A	0.5583	−0.0721	0.5061	0.202*	
H19B	0.6136	−0.1522	0.5027	0.202*	
H19C	0.5372	−0.1487	0.4179	0.202*	

### Atomic displacement parameters (Å^2^)

**Table:**

	*U*^11^	*U*^22^	*U*^33^	*U*^12^	*U*^13^	*U*^23^
O1	0.0501 (10)	0.0581 (10)	0.0683 (11)	0.0038 (8)	−0.0064 (8)	−0.0100 (8)
O2	0.0362 (9)	0.0680 (11)	0.0886 (12)	0.0093 (7)	0.0209 (8)	0.0083 (9)
O3	0.0506 (9)	0.0445 (9)	0.0687 (10)	0.0078 (7)	0.0133 (8)	0.0154 (8)
C1	0.0392 (12)	0.0532 (13)	0.0402 (12)	0.0006 (10)	0.0125 (10)	−0.0089 (10)
C2	0.0412 (12)	0.0520 (13)	0.0357 (11)	0.0039 (10)	0.0150 (10)	−0.0004 (10)
C3	0.0450 (12)	0.0457 (12)	0.0434 (12)	0.0003 (9)	0.0179 (10)	0.0027 (10)
C4	0.0370 (11)	0.0462 (12)	0.0406 (11)	0.0020 (9)	0.0149 (9)	−0.0023 (9)
C5	0.0405 (12)	0.0485 (13)	0.0403 (11)	0.0072 (9)	0.0107 (10)	0.0008 (10)
C6	0.0426 (12)	0.0442 (12)	0.0424 (12)	0.0030 (9)	0.0146 (10)	−0.0024 (10)
C7	0.0490 (14)	0.0466 (13)	0.0623 (15)	0.0011 (10)	0.0071 (12)	−0.0023 (11)
C8	0.081 (2)	0.0697 (17)	0.105 (2)	−0.0119 (14)	0.0392 (17)	0.0075 (16)
C9	0.098 (2)	0.0500 (15)	0.091 (2)	−0.0007 (14)	−0.0152 (17)	0.0087 (14)
C10	0.083 (2)	0.0578 (16)	0.0886 (19)	0.0071 (13)	0.0263 (16)	−0.0202 (14)
C11	0.0536 (14)	0.0575 (14)	0.0390 (12)	0.0061 (10)	0.0137 (10)	0.0040 (10)
C12	0.0547 (16)	0.0748 (17)	0.0890 (19)	0.0154 (12)	0.0204 (14)	0.0210 (15)
C13	0.0780 (19)	0.0861 (19)	0.0679 (17)	−0.0075 (15)	0.0115 (14)	0.0331 (15)
C14	0.146 (3)	0.114 (2)	0.0415 (15)	0.039 (2)	0.0247 (17)	0.0077 (15)
C15	0.0357 (11)	0.0466 (12)	0.0462 (12)	0.0021 (9)	0.0135 (9)	0.0023 (10)
C16	0.0318 (15)	0.0369 (15)	0.0434 (16)	0.000	0.0079 (13)	0.000
C17	0.0389 (13)	0.0398 (12)	0.0529 (13)	−0.0006 (9)	0.0118 (10)	−0.0039 (10)
C18	0.080 (2)	0.0642 (17)	0.088 (2)	0.0283 (14)	0.0186 (16)	0.0248 (15)
C19	0.120 (3)	0.099 (2)	0.193 (4)	0.031 (2)	0.066 (3)	0.083 (3)

### Geometric parameters (Å, °)

**Table:**

O1—C1	1.381 (2)	C10—H10B	0.9600
O1—H1	0.803 (16)	C10—H10C	0.9600
O2—C17	1.196 (2)	C11—C14	1.528 (3)
O3—C17	1.336 (2)	C11—C12	1.532 (3)
O3—C18	1.459 (3)	C11—C13	1.535 (3)
C1—C6	1.403 (3)	C12—H12A	0.9600
C1—C2	1.407 (3)	C12—H12B	0.9600
C2—C3	1.389 (3)	C12—H12C	0.9600
C2—C11	1.540 (3)	C13—H13A	0.9600
C3—C4	1.380 (3)	C13—H13B	0.9600
C3—H3A	0.9300	C13—H13C	0.9600
C4—C5	1.388 (3)	C14—H14A	0.9600
C4—C15	1.516 (3)	C14—H14B	0.9600
C5—C6	1.390 (3)	C14—H14C	0.9600
C5—H5A	0.9300	C15—C16	1.554 (2)
C6—C7	1.547 (3)	C15—H15A	0.9700
C7—C9	1.526 (3)	C15—H15B	0.9700
C7—C8	1.527 (4)	C16—C17^i^	1.526 (2)
C7—C10	1.538 (3)	C16—C17	1.526 (2)
C8—H8A	0.9600	C16—C15^i^	1.554 (2)
C8—H8B	0.9600	C18—C19	1.442 (4)
C8—H8C	0.9600	C18—H18A	0.9700
C9—H9A	0.9600	C18—H18B	0.9700
C9—H9B	0.9600	C19—H19A	0.9600
C9—H9C	0.9600	C19—H19B	0.9600
C10—H10A	0.9600	C19—H19C	0.9600
			
C1—O1—H1	110.7 (19)	C12—C11—C2	110.30 (17)
C17—O3—C18	115.76 (19)	C13—C11—C2	111.76 (18)
O1—C1—C6	115.80 (19)	C11—C12—H12A	109.5
O1—C1—C2	121.62 (18)	C11—C12—H12B	109.5
C6—C1—C2	122.53 (18)	H12A—C12—H12B	109.5
C3—C2—C1	116.16 (18)	C11—C12—H12C	109.5
C3—C2—C11	121.36 (18)	H12A—C12—H12C	109.5
C1—C2—C11	122.48 (18)	H12B—C12—H12C	109.5
C4—C3—C2	123.98 (19)	C11—C13—H13A	109.5
C4—C3—H3A	118.0	C11—C13—H13B	109.5
C2—C3—H3A	118.0	H13A—C13—H13B	109.5
C3—C4—C5	117.11 (18)	C11—C13—H13C	109.5
C3—C4—C15	121.26 (18)	H13A—C13—H13C	109.5
C5—C4—C15	121.46 (17)	H13B—C13—H13C	109.5
C4—C5—C6	123.08 (18)	C11—C14—H14A	109.5
C4—C5—H5A	118.5	C11—C14—H14B	109.5
C6—C5—H5A	118.5	H14A—C14—H14B	109.5
C5—C6—C1	116.87 (19)	C11—C14—H14C	109.5
C5—C6—C7	121.54 (17)	H14A—C14—H14C	109.5
C1—C6—C7	121.56 (18)	H14B—C14—H14C	109.5
C9—C7—C8	107.1 (2)	C4—C15—C16	116.22 (15)
C9—C7—C10	107.7 (2)	C4—C15—H15A	108.2
C8—C7—C10	109.6 (2)	C16—C15—H15A	108.2
C9—C7—C6	111.54 (18)	C4—C15—H15B	108.2
C8—C7—C6	111.67 (18)	C16—C15—H15B	108.2
C10—C7—C6	109.11 (19)	H15A—C15—H15B	107.4
C7—C8—H8A	109.5	C17^i^—C16—C17	106.2 (2)
C7—C8—H8B	109.5	C17^i^—C16—C15^i^	110.79 (10)
H8A—C8—H8B	109.5	C17—C16—C15^i^	108.55 (11)
C7—C8—H8C	109.5	C17^i^—C16—C15	108.55 (11)
H8A—C8—H8C	109.5	C17—C16—C15	110.79 (10)
H8B—C8—H8C	109.5	C15^i^—C16—C15	111.9 (2)
C7—C9—H9A	109.5	O2—C17—O3	123.65 (19)
C7—C9—H9B	109.5	O2—C17—C16	126.01 (18)
H9A—C9—H9B	109.5	O3—C17—C16	110.34 (17)
C7—C9—H9C	109.5	C19—C18—O3	108.3 (3)
H9A—C9—H9C	109.5	C19—C18—H18A	110.0
H9B—C9—H9C	109.5	O3—C18—H18A	110.0
C7—C10—H10A	109.5	C19—C18—H18B	110.0
C7—C10—H10B	109.5	O3—C18—H18B	110.0
H10A—C10—H10B	109.5	H18A—C18—H18B	108.4
C7—C10—H10C	109.5	C18—C19—H19A	109.5
H10A—C10—H10C	109.5	C18—C19—H19B	109.5
H10B—C10—H10C	109.5	H19A—C19—H19B	109.5
C14—C11—C12	111.0 (2)	C18—C19—H19C	109.5
C14—C11—C13	106.6 (2)	H19A—C19—H19C	109.5
C12—C11—C13	105.9 (2)	H19B—C19—H19C	109.5
C14—C11—C2	111.06 (18)		
			
O1—C1—C2—C3	−178.03 (19)	C1—C6—C7—C10	−59.1 (3)
C6—C1—C2—C3	4.5 (3)	C3—C2—C11—C14	−126.1 (3)
O1—C1—C2—C11	2.3 (3)	C1—C2—C11—C14	53.5 (3)
C6—C1—C2—C11	−175.11 (18)	C3—C2—C11—C12	110.4 (2)
C1—C2—C3—C4	0.2 (3)	C1—C2—C11—C12	−69.9 (3)
C11—C2—C3—C4	179.83 (18)	C3—C2—C11—C13	−7.1 (3)
C2—C3—C4—C5	−3.3 (3)	C1—C2—C11—C13	172.5 (2)
C2—C3—C4—C15	−178.59 (19)	C3—C4—C15—C16	−81.2 (2)
C3—C4—C5—C6	1.8 (3)	C5—C4—C15—C16	103.7 (2)
C15—C4—C5—C6	177.16 (19)	C4—C15—C16—C17^i^	163.70 (16)
C4—C5—C6—C1	2.5 (3)	C4—C15—C16—C17	47.5 (2)
C4—C5—C6—C7	−175.45 (19)	C4—C15—C16—C15^i^	−73.76 (15)
O1—C1—C6—C5	176.62 (18)	C18—O3—C17—O2	−2.7 (3)
C2—C1—C6—C5	−5.8 (3)	C18—O3—C17—C16	176.81 (18)
O1—C1—C6—C7	−5.4 (3)	C17^i^—C16—C17—O2	129.7 (2)
C2—C1—C6—C7	172.14 (19)	C15^i^—C16—C17—O2	10.6 (3)
C5—C6—C7—C9	−0.2 (3)	C15—C16—C17—O2	−112.6 (2)
C1—C6—C7—C9	−178.0 (2)	C17^i^—C16—C17—O3	−49.75 (12)
C5—C6—C7—C8	−120.0 (2)	C15^i^—C16—C17—O3	−168.89 (16)
C1—C6—C7—C8	62.1 (3)	C15—C16—C17—O3	67.9 (2)
C5—C6—C7—C10	118.7 (2)	C17—O3—C18—C19	178.4 (3)

Symmetry codes: (i) −*x*+1, *y*, −*z*+1/2.

### Hydrogen-bond geometry (Å, °)

**Table:**

*D*—H···*A*	*D*—H	H···*A*	*D*···*A*	*D*—H···*A*
O1—H1···O2^ii^	0.80 (2)	2.38 (2)	2.996 (2)	134 (2)
C12—H12C···O2^ii^	0.96	2.46	3.398 (3)	167

Symmetry codes: (ii) −*x*+3/2, −*y*+1/2, −*z*+1.
